# Multiproxy paleoceanographic study from the western Barents Sea reveals dramatic Younger Dryas onset followed by oscillatory warming trend

**DOI:** 10.1038/s41598-020-72747-4

**Published:** 2020-09-24

**Authors:** Magdalena Łącka, Danuta Michalska, Joanna Pawłowska, Natalia Szymańska, Witold Szczuciński, Matthias Forwick, Marek Zajączkowski

**Affiliations:** 1 Institute of Oceanology, Polish Academy of Sciences, Powstańców Warszawy 55, 81-712 Sopot, Poland; 2 Institute of Geology, Adam Mickiewicz University in Poznań, Bogumiła Krygowskiego 12, 61-680 Poznań, Poland; 3 Department of Geosciences, UiT The Arctic University of Norway in Tromsø, N-9037 Tromsø, Norway

**Keywords:** Climate sciences, Palaeoceanography

## Abstract

The Younger Dryas (YD) is recognized as a cool period that began and ended abruptly during a time of general warming at the end of the last glacial. New multi-proxy data from a sediment gravity core from Storfjordrenna (western Barents Sea, 253 m water depth) reveals that the onset of the YD occurred as a single short-lived dramatic environment deterioration, whereas the subsequent warming was oscillatory. The water masses in the western Barents Sea were likely strongly stratified at the onset of the YD, possibly due to runoff of meltwater combined with perennial sea-ice cover, the latter may last up to several decades without any brake-up. Consequently, anoxic conditions prevailed at the bottom of Storfjordrenna, leading to a sharp reduction of benthic biota and the appearance of vivianite microconcretions which formation is favoured by reducing conditions. While the anoxic conditions in Storfjordrenna were transient, the unfavorable conditions for benthic foraminifera lasted for c. 1300 years. We suggest that the Pre-Boreal Oscillation, just after the onset of the Holocene, may have been a continuation of the oscillatory warming trend during the YD.

## Introduction

The Atlantic Meridional Overturning Circulation (AMOC) transports heat and salt northwards throughout the southern and northern Atlantic Ocean. Numerous climate predictions using numerical and theoretical models of ocean circulation suggest that the AMOC will weaken over the coming century due to glacial meltwater runoff and decreases in sea ice cover under global warming^1^.

One of the most striking examples of AMOC weakening from the geological past is the Bølling-Allerød (B-A)–Younger Dryas (YD; c. 12.8–11.7 ka BP) transition. The YD constituted a return to near-glacial conditions after the Earth’s climate began to shift from a cold glacial world to a warmer interglacial state at the end of the last glacial. It is generally accepted that the YD cold event resulted from a slowdown in the AMOC^2^. However, its consequences^3^ as well as its other hypothetic oceanic^2^, extraterrestrial^4^, volcanic^5^, and atmospheric^6^ causes are still debated.

Numerous marine sedimentary records attest that the northern hemisphere was subjected to rapid cooling over circa 1000 years during the YD^7,8^. As earlier investigations focused mainly on overviews of Late Glacial and Holocene paleoceanography of the Nordic Seas, the YD interval in marine records was presented in low temporal resolution (several hundreds of years). Although environmental variability during the YD is documented in records from the northern Atlantic Ocean^9–11^, high-resolution records from the northern part of the Nordic Seas—where the effects of ongoing global climate change are most pronounced^12^—remain absent.

**Figure 1:**
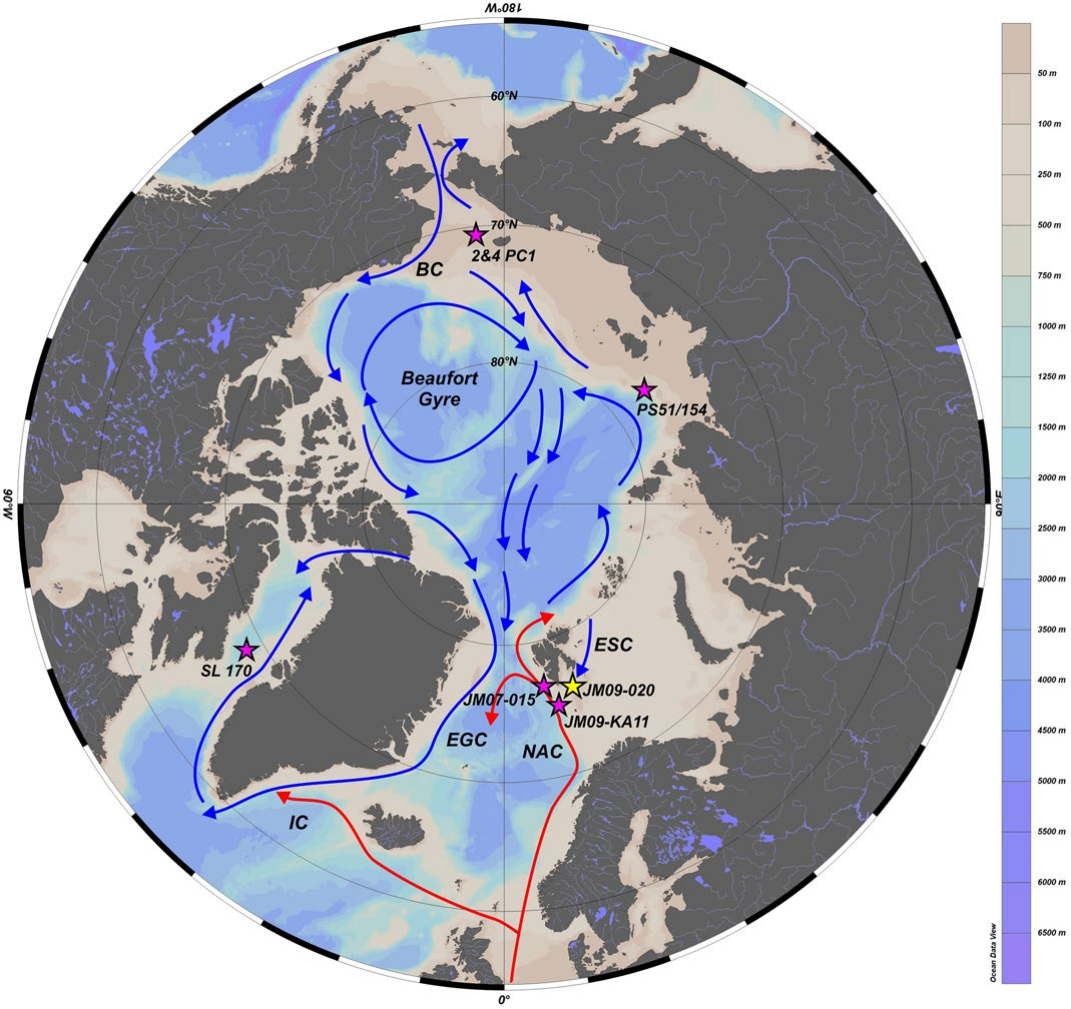
Map of the Arctic Ocean (from Ocean Data View, version ODV 5.2.1, https://odv.awi.de/^14^) showing present-day surface-water circulation in the North Atlantic and Arctic Oceans after Armitage et al.^15^ with marked locations of the core JM09-020-GC (this study; yellow star), and other cores discussed in this paper (violet stars): SL 170^16^, 2&4 PC1^17^, PS51/154^18^, JM07-015^19^, JM09-KA11^20,21^. *NAC* North Atlantic Current, *ESC* East Spitsbergen Current, *EGC* East Greenland Current, *IC* Irminger Current, *BC* Bering Current.

An earlier study of sediment gravity core JM09-020-GC from Storfjordrenna (western Barents Sea; Fig. 1) revealed that the YD was not uniformly cold as had earlier been proposed and that at least some warmer periods occurred^13^. In this paper, we present a new multi-proxy record of the YD from core JM09-020-GC with higher temporal resolution than that presented in Łącka et al.^13^ consisting of sedimentological (ice-rafted debris counts), mineralogical (analyses of vivianite), micropaleontological (benthic foraminifera counts) and geochemical (Mn/Fe, oxygen stable isotopes, composition of microconcretions) analyses and we compare our results to other paleoclimatic records. Our new findings, as well as new data concerning the Arctic Ocean circulation at that time, prompted us to resume the discussion on the YD trigger and its evolution. The aim of this study is to provide a more detailed understanding of the oceanographic variability that occurred in the western Barents Sea during this stadial.

## Results and discussion

### Onset of YD

**Figure 2:**
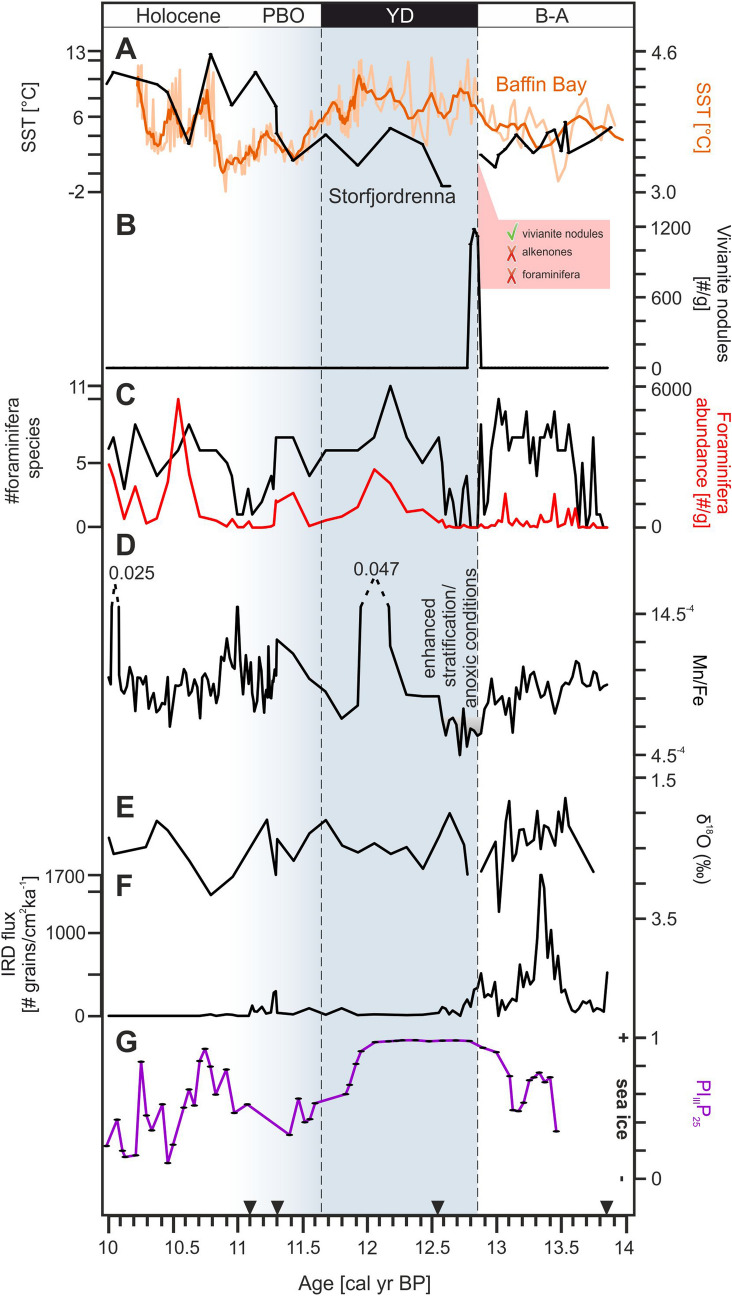
Proxy data from core JM09-020-GC with relevant paleoclimate data plotted versus age. Chronostratigraphic units are indicated: B-A-Bølling-Allerød; YD-Younger Dryas; PBO-Pre-Boreal Oscillation. (**A**) alkenone-based SST^22^ (left scale; black line) and SST from central Baffin Bay^16^ (right scale; light orange line) with five-point running average (right scale; dark orange line); (**B**) content of vivianite nodules; (**C**) total benthic foraminiferal abundance (right scale; red line) and foraminiferal biodiversity expressed as number of species (left scale; black line); (**D**) Mn/Fe ratio; (**E**) *Elphidium clavatum* δ^18^O; (**F**) IRD flux; (**G**) Sea ice proxy P_III_P_25_ from Kveithola Trough^20^. The black triangles on the x-axis denote the obtained AMS ^14^C datings converted to calibrated radiocarbon ages.

**Figure 3:**
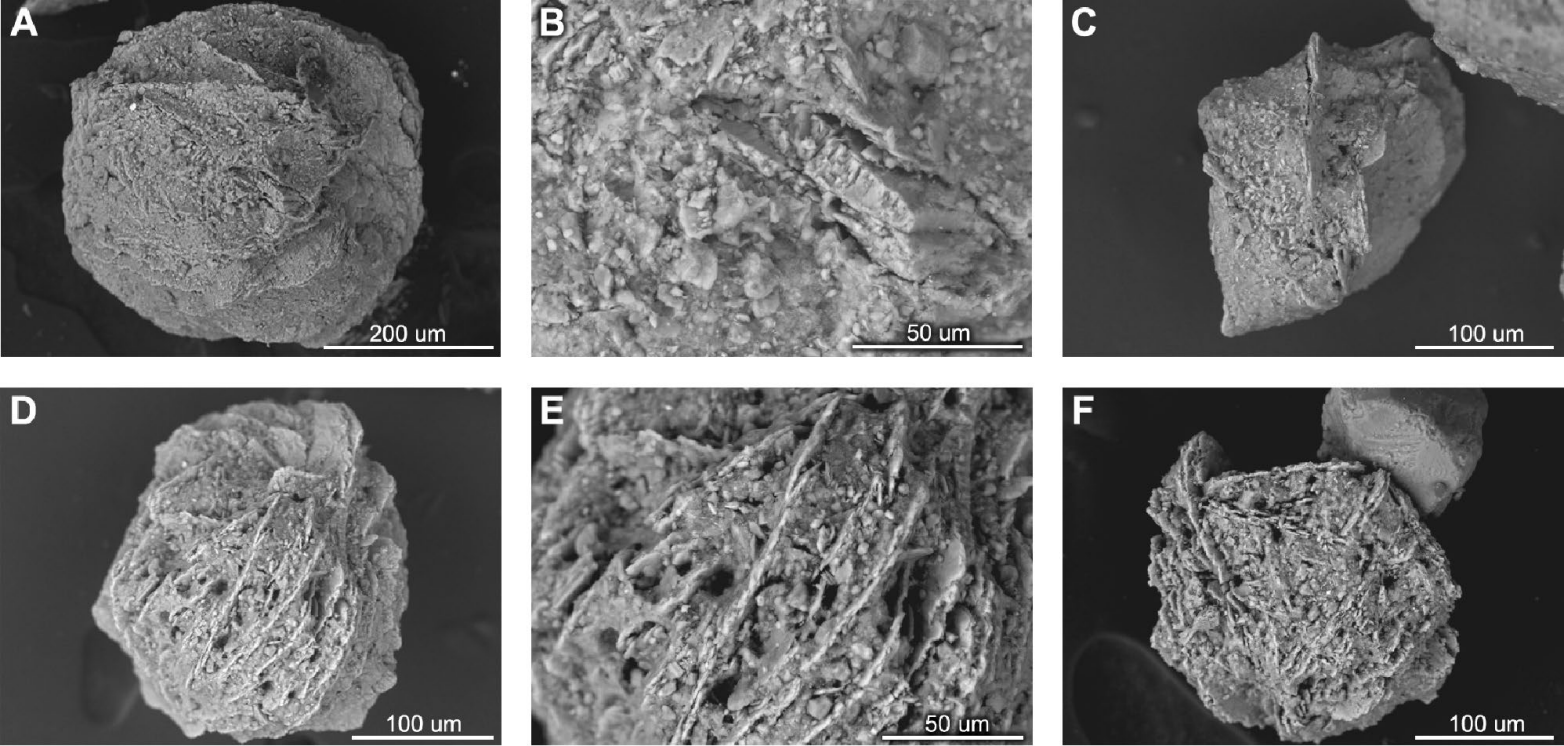
SEM images of vivianite microconcretions found in samples taken from between 293 and 295 cm core depth. (**A**) Diameter 0.443 mm; (**B**) magnification × 850 of the microconcretion presented in (**A**); (**C**) fusiform microconcretion, long axis 0.255 mm; (**D**) diameter 0.257 mm; (**E**) magnification × 700 of the microconcretion presented in (**D**); (**F**) longer axis 0.224 mm.

Pronounced changes in sedimentological, geochemical, and foraminiferal compositions occurred in the sediment core section dated to the likely transition from the B-A to the YD around 12.85 ka BP in Storfjordrenna (Fig. 2). At the very beginning of the YD, foraminifera were absent and ice-rafted debris (IRD) flux was low, the latter likely resulting from suppressed iceberg drift due to perennial sea ice^13^. Numerous vivianite microconcretions (between 10,500 and 11,800 nodules g^−1^) were found in three adjacent 1-cm thick sediment samples (293–295 cm) dated to approximately 12.80–12.85 ka BP (Supplementary Material; Fig. S3). The found vivianite nodules varied in size (200–450 µm; average size 230 µm) and shape (spherical, fusiform, oval). However, all microconcretions had a “desert rose”-like appearance (Fig. 3) and intense purple color. The identification of vivianite was confirmed by X-ray diffraction and semiquantitative geochemical composition analyses using scanning electron microscope energy dispersive spectroscopy (SEM–EDS; Supplementary Material; Figs. S1 and S2).

**Figure 4:**
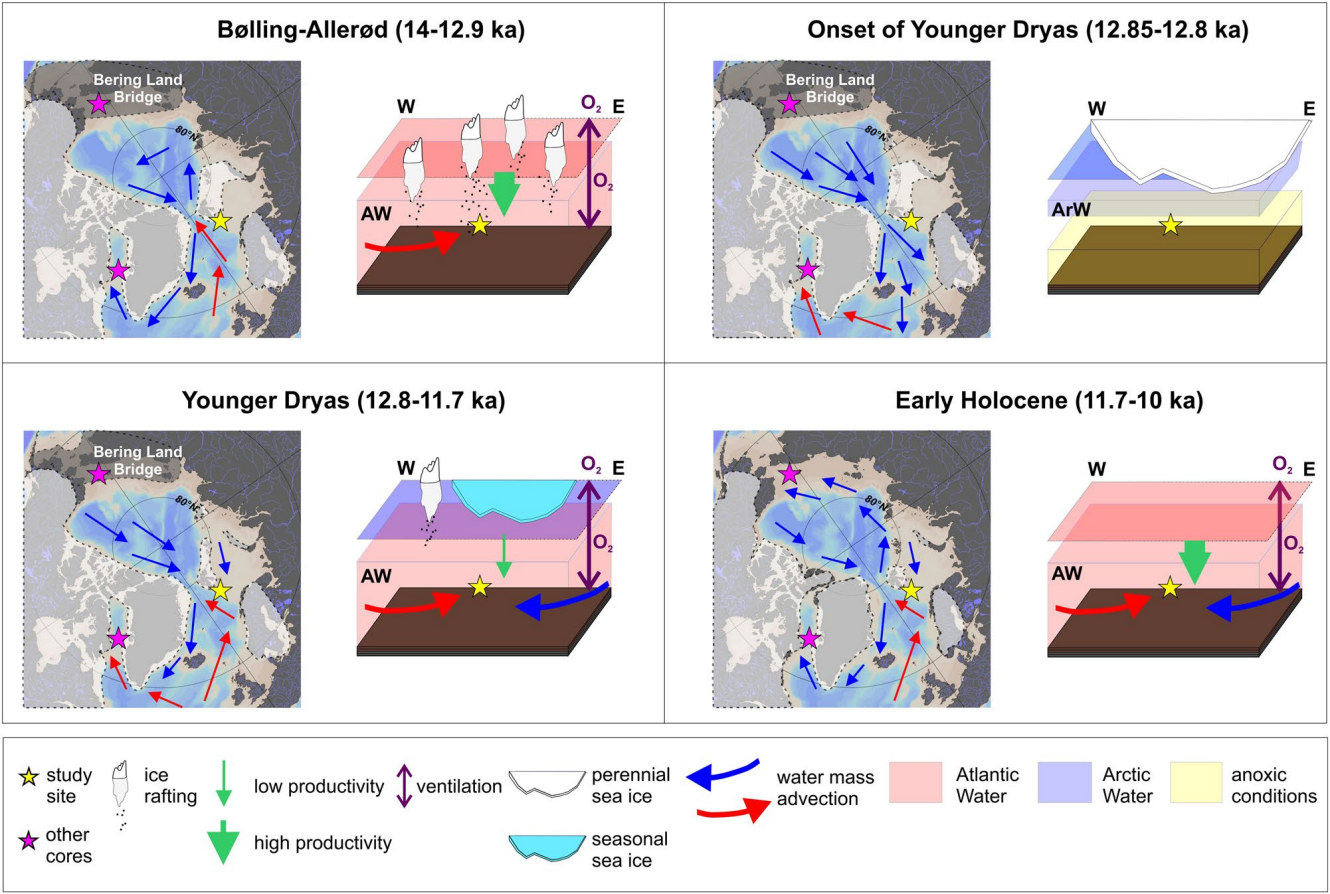
Schematic summary of the environmental and climatic changes in the North Atlantic and Arctic Ocean region during 14–10 ka along a tentative transect across core JM09-020-GC (study site), marked with the yellow star (map retrieved from Ocean Data View, version ODV 5.2.1, https://odv.awi.de/)^14^. See Fig. 1 for the detailed description of the other marked sediment cores. *AW* Atlantic Water, *ArW* Arctic Water. The ice sheets extent are adapted from Petrini et al.^27^, Heinemann et al.^28^ and Hughes et al.^29^.

Vivianite (hydrated iron phosphate) is a mineral formed in various aquatic environments^23^. In marine sediments, it crystalizes in reducing conditions with sulfide-depleted porewaters that are rich in both Fe^2+^ and PO_4_, therefore its presence may reflect anoxic bottom waters upon sediment deposition^23–25^. As manganese is more soluble than iron under reducing conditions^26^, the low Mn/Fe ratio (Fig. 2D) supports the existence of low oxygen levels in Storfjordrenna bottom sediments at the onset of the YD (Fig. 4). The prolonged oxygen depletion most likely resulted from reduced atmosphere–ocean gas exchange due to perennial sea-ice cover and, thus, very limited vertical (e.g., brine release during sea-ice formation), as well as reduced lateral water movement (e.g., limited advection of AW; Fig. 4). Furthermore, the remnants of the Svalbard-Barents Sea ice sheet (SBIS) formed a partly protecting embayment around the core site^13^, potentially supporting anoxic conditions as vertical water mixing may have been limited due to strong water stratification caused by a brackish water surface layer deriving from meltwater runoff. The absence of foraminifera likely results from bottom-water anoxia and limited food supply due to low primary production (reflected by a lack of alkenones signals^22^; Fig. 2A).

Vivianite in marine deposits may be allochthonous or authigenic in origin^25^. The vivianite nodules found at the YD onset in Storfjordrenna are interpreted as authigenic in origin due to the absence of abrasion and dissolution features on mineral surfaces, which could suggest their formation in different environments (e.g., freshwater) and/or long transport. Vivianite microconcretions from Arctic sediments have hitherto been reported exclusively from the Laptev Sea, where their appearance accompanied by rhodochrosite concretions (not present in our study site) has been linked to enhanced water stratification caused by surface water freshening and the development of a thick ice cover for large portions of the year^18^. Taldenkova et al.^18^ assumed that water freshening was largely due to glacial meltwater input and proposed that concretion formation reflects meltwater events. A similar interpretation may apply for Storfjordrenna. Increased δ^18^O just after the onset of the YD may reflect high-saline water near the bottom (Fig. 2E). However, the isotopic record reflects bottom waters and not of surface waters potentially affected by meltwater release; furthermore isotopic data are lacking for the actual vivianite-rich layers (absence of foraminifera). The onset of YD occurred just after a long warming trend (B-A; Figs. 2 and 4) that likely led to enhanced meltwater production from remnants of the nearby SBIS^12^. Finally, the formation of vivianite concretions found in Storfjordrenna required numerous active forms of iron, known to be released in large amounts by glacial meltwaters^30,31^.

The appearance of vivianite in three adjacent sediment samples deposited likely in the earliest YD confirms a dramatic transition from ameliorated conditions during B-A with constant AW inflow^13,22^ to anoxic conditions that likely resulted from enhanced stratification, perennial sea-ice cover, and reduced advection of AW (Fig. 4). The YD is reflected in multiple records from the northeastern North Atlantic Ocean^7,8,19^. For instance, in Kveithola Trough located south of Storfjordrenna, the sea-ice proxy P_III_IP_25_ (Fig. 2G) pointed to long seasonal sea ice cover thorough YD—characteristic of the northern Barents Sea in modern times^20,21^. However, such pronounced environmental changes at the YD onset, as identified in our study, remain undocumented. The absence of vivianite microconcretions in earlier studies may be related to various factors: (1) studies performed hitherto lack sufficient temporal resolution; (2) whereas some studies reflected open-ocean conditions, Storfjordrenna was a semi-enclosed glacial bay at that time^13,29^, permitting bottom-water mass stagnation, strong stratification, and large supplies of Fe-oxides in meltwater—factors that are essential for vivianite nodule formation^32^. However, the likely anoxic conditions in near-bottom waters in the western Barents Sea at that time are also confirmed by Sternal et al.^19^ in a study from the continental shelf offshore southwestern Svalbard reporting high authigenic pyrite, organic carbon and sulphur content around the onset of the YD. Our observation of vivianite is the second ever observation of vivianite in the sediments of the Arctic shelf areas. Therefore still more studies are needed to better understand its formation in a generally well-oxygenated environment.

Our data suggest that perennial sea-ice cover possibly prevailed at the onset of the YD. This may be related to a temporary AMOC slowdown or reorganization of oceanographic currents in the northern Atlantic Ocean and a consequent reduction in AW supply to the Nordic Seas (Fig. 4). At that time, the Bering Strait was still closed^17^ or partly open^33^, so the only effective freshwater outflow from Siberian rivers and melting ice caps was through the Fram Strait and the Barents Sea. According to empirical and modeling data^16,34^, a warming in the northwestern part of the North Atlantic occurred at the beginning of the YD. Warmer sea-surface conditions in Baffin Bay throughout the YD (Figs. 1, 2A) are related to the intensified inflow of AW carried by the Irminger Current at that time^16^. Whereas Baffin Bay is presently covered by sea ice for most of the year, the western Barents Sea experiences an intensified “atlantification” with constantly declining sea ice and increasing sea-surface temperature (SST)^35^. The YD conditions, in contrast to the environment in the modern North Atlantic Ocean, suggest that the lid of the freshwater from Siberian rivers and melting ice sheets possibly contributed to the reduction of the inflow of AW to the eastern part of the North Atlantic Ocean, forcing these water masses to spread along the eastern coast of North America (Fig. 4). This scenario supports the results from Rainsley et al.’s model^34^. However, their model underestimates the impact of the meltwater on the hydrography of the North Atlantic Ocean. According to the model, AW reached the British Islands during the first part of the YD, whereas the empirical data indicate pronounced cooling during the YD in Wales, Ireland, and northern England^36^, suggesting that the surface meltwater layer could reduce the climatic impact of the AW inflow.

### Development of YD

Shortly after the anoxic period (c. 12.7 ka BP), opportunistic benthic foraminifera, such as *E. clavatum* and *Cassidulina reniforme*, appeared in Storfjordrenna (Supplementary Material; Fig. S4). However, they were low in quantity (Fig. 2C). This may indicate the presence of seasonal sea ice in Storfjordrenna, enabling the growth of opportunistic foraminifera species adapted to low productivity conditions and strong water stratification limiting water and gas exchange, as suggested by the low Mn/Fe values (Figs. 2D and 4).

Rapid increases in foraminiferal biodiversity and total foraminiferal abundance occurred around 12.4 ka BP. These were associated with an increase in the Mn/Fe ratio, indicating normal oxic conditions^26^ (Fig. 2D). The changes were associated with higher SST and heavier δ^18^O indicating a restored AW inflow (Figs. 2E and 4). However, a decimation of foraminiferal assemblage with *E. clavatum* representing almost 90% of the total count (Supplementary Material; Fig. S4) and a decrease of SST occurred around 11.9 ka BP (Fig. 2A).

The earliest Holocene in Storfjordrenna (c. 11.45–11.3 ka) is marked by a pronounced increase in ice rafting, greater foraminifera abundance, and concomitant higher foraminiferal biodiversity likely corresponding to the increasing SST (Figs. 2 and 4). However, the benthic foraminifera fauna was still dominated by species connected with Arctic Water (e.g., *E. clavatum*)^13^. The bottom waters were likely much better ventilated than during the early and late YD (higher Mn/Fe). By contrast, the beginning of the Holocene in Baffin Bay was characterized by a marked decrease in SST^16^ (Fig. 2A). Benthic foraminifera in Storfjordrenna declined sharply around 11.3 ka BP, accompanied by an increase in δ^18^O, similar to the onset of the YD. We relate this change to the Pre-Boreal Oscillation, a cold event documented in multiple records from the North Atlantic region^37,38^ and linked to meltwater delivery and AMOC weakening^39^. A short-lived increase in SST occurred simultaneously in Baffin Bay^16^. This may result from a mechanism similar to that discussed in relation to the YD.

The SST in Storfjordrenna increased sharply at c. 11.15 cal year BP, synchronously with the opening of the Bering Strait^17^ and the further decline in SST in Baffin Bay (Figs. 2A and 4). The modern circulation pattern in the Northern Hemisphere was established at that time.

## Conclusions

We have demonstrated that the onset of the YD in the western Barents Sea was likely much more dramatic than is generally inferred. Because the Bering Strait was locked or partly opened at that time, the only effective meltwater outflow from the Arctic Ocean was through the Fram Strait and the Barents Sea (Fig. 4). Perennial sea-ice cover in the western Barents Sea was most likely formed as a consequence of the slowdown and westward migration of the AMOC. Along with the likely significant glacial meltwater supply, the strong stratification in Storfjordrenna supported anoxic seafloor conditions for approximately a century at the onset of YD. Throughout YD, several similar abrupt coolings occurred synchronously with SST warming in Baffin Bay. We suggest that the last of such periods was the Pre-Boreal Oscillation just after the onset of the Holocene. However, the scarcity of high-resolution and well-dated records from the discussed time periods and inevitable dating uncertainties, limit our understanding of the overall oceanography during these abrupt reversals. Our study shows the relevance of high temporal resolution studies of marine sediment records to identify, as well as to understand the magnitudes and consequences of short-lasting environmental changes.

## Methods

### Coring and sampling

Sediment gravity core JM09-020-GC was retrieved with R/V Jan Mayen (now R/V Helmer Hanssen) from Storfjordrenna (western Barents Sea; 76° 31,489′ N, 19° 69,957′ E) in November 2009 from 253 m water depth^13^ (Fig. 1). The studied interval (252–301 cm depth below seafloor) was sampled at 1 cm intervals. X-radiographs and digital images were collected from half-core sections (Supplementary Material; Fig. S3).

### Chronology

The chronology for this study is based on Łącka et al.^13^ supplemented with three additional accelerator mass spectrometer (AMS) ^14^C measurements (Table 1). The dates were converted into calibrated ages using the Marine13 calibration curve^40^ and ΔR of 105 ± 24^41^ in the CALIB 7.1 program^42^. The age model is based on the assumption of linear sediment accumulation rates between data points (Supplementary Material; Fig. S3). The highest probability peaks from the calibrated age ranges were used as input values for the model. Figure 2 shows the age control points used to produce the age model. Measurements were performed in the Poznań Radiocarbon Laboratory, which is equipped with a 1.5 SDH-Pelletron Model “Compact Carbon AMS”^43,44^. The surface layer of bivalves shells was scraped off to avoid contamination with younger carbonate encrustation.

**Table 1:** AMS ^14^C dates and calibrated ages.

Sample no.	Depth (cm)	Lab no.	Raw AMS ^14^C BP	Calibrated years BP ± 2σ	Cal year BP used in age model	Dated material
St 20251/252/253*	252	Poz-46964	10,200 ± 60	10,895–11,230	11,100	*Thracia* sp 0.5 mgC
St 20273/274	273.5	Poz-73949	11,440 ± 90	11,152–11,789	11,300	*E.clavatum* 0.09 mgC
St 20283/284	283.5	Poz-71208	11,090 ± 110	12,116–12,710	12,550	Bivalvia shell 0.07 mgC
St 20332	331.5	Poz-17182	12,650 ± 80	13,802–14,252	13,850	Bivalvia shell 0.3 mgC

The previously published data^13^ are marked with asterisks.

### Analyses of benthic foraminifera assemblages

The samples were washed on a 100 μm mesh-size sieve. Throughout the late Pleistocene/early Holocene interval abundances of foraminifera are low. 66 of the 82 studied samples contained < 300 benthic foraminifer shells. Samples with significantly more than 300 specimens were split using a traditional hand-splitter until a suitable aliquot remained. Picked foraminifera were identified under a stereo-microscope. Classification and identification were carried out in accordance with literature on subfossil Arctic foraminifera. In total, 22 benthic calcareous foraminifera species were identified.

### Oxygen stable isotopes analysis

Oxygen stable isotope compositions of tests of the infaunal foraminifer species *Elphidium clavatum* were determined at the Department of Geological Sciences, University of Florida (Florida, USA). All values are calibrated to the PeeDee Belemnite (PDB) scale and corrected for ice volume changes^45^.

### Ice-rafted debris

The ice-rafted debris (IRD; grains > 500 µm) were counted under a stereo-microscope and expressed as flux values (number of grains cm^−2^ ka^−1^) using the sediment accumulation rate.

### X-ray fluorescence scanning

Qualitative element-geochemical measurements were performed at Department of Geology (now Department of Geosciences), UiT with an Avaatech X-ray fluorescence (XRF) core scanner using the following settings: 10 kV, 1000 µA, 10-s measuring time, and no filter. The manganese/iron ratio (Mn/Fe) was used as an indicator of reducing conditions^26^.

### Vivianite

The morphologies and semiquantitative chemical compositions of concretions were studied with a Hitachi S-3700N Scanning Electron Microscope with Energy Dispersive Spectrometer (SEM–EDS; 30 Pa, 20 kv, BSE) at Faculty of Geographical and Geological Sciences in Poznań. The X-ray diffraction (XRD) analysis of powdered samples were run on a ARL X’tra [theta–theta goniometer; Cu Kα radiation; Peltier cooled Si(Li) solid-state detector] from Thermo Electron at Institute of Geology, Adam Mickiewicz University, Poznań.

## Supplementary information

Supplementary Figures.

## Data Availability

All data presented in this paper are available at open database for Earth and Environmental Science PANGAEA (https://doi.pangaea.de/10.1594/PANGAEA.917645).
