# Supplementary material for: Multiproxy paleoceanographic study from the western Barents Sea reveals dramatic Younger Dryas onset followed by oscillatory warming trend
Source: Sci Rep. 2020 Sep 24;10:15667. doi: 10.1038/s41598-020-72747-4 (PMC7515869; doi:10.1038/s41598-020-72747-4)
Supplement: Supplementary file 1 — Supplementary Figures. [file 41598_2020_72747_MOESM1_ESM.docx]

Supplementary material to

**Multiproxy paleoceanographic study from the western Barents Sea reveals dramatic Younger Dryas onset followed by oscillatory warming trend**

Magdalena Łącka^1^*, Danuta Michalska^2^, Joanna Pawłowska^1^, Natalia Szymańska^1^, Witold Szczuciński^2^, Matthias Forwick^3^ and Marek Zajączkowski^1^

^1^Institute of Oceanology, Polish Academy of Sciences, Powstańców Warszawy 55, 81-712 Sopot, Poland

^2^Institute of Geology, Adam Mickiewicz University in Poznań, Bogumiła Krygowskiego 12, 61-680 Poznań, Poland

^3^Department of Geosciences, UiT The Arctic University of Norway in Tromsø, N-9037 Tromsø, Norway

*corresponding author [mlacka@iopan.gda.pl](mailto:mlacka@iopan.gda.pl)

**
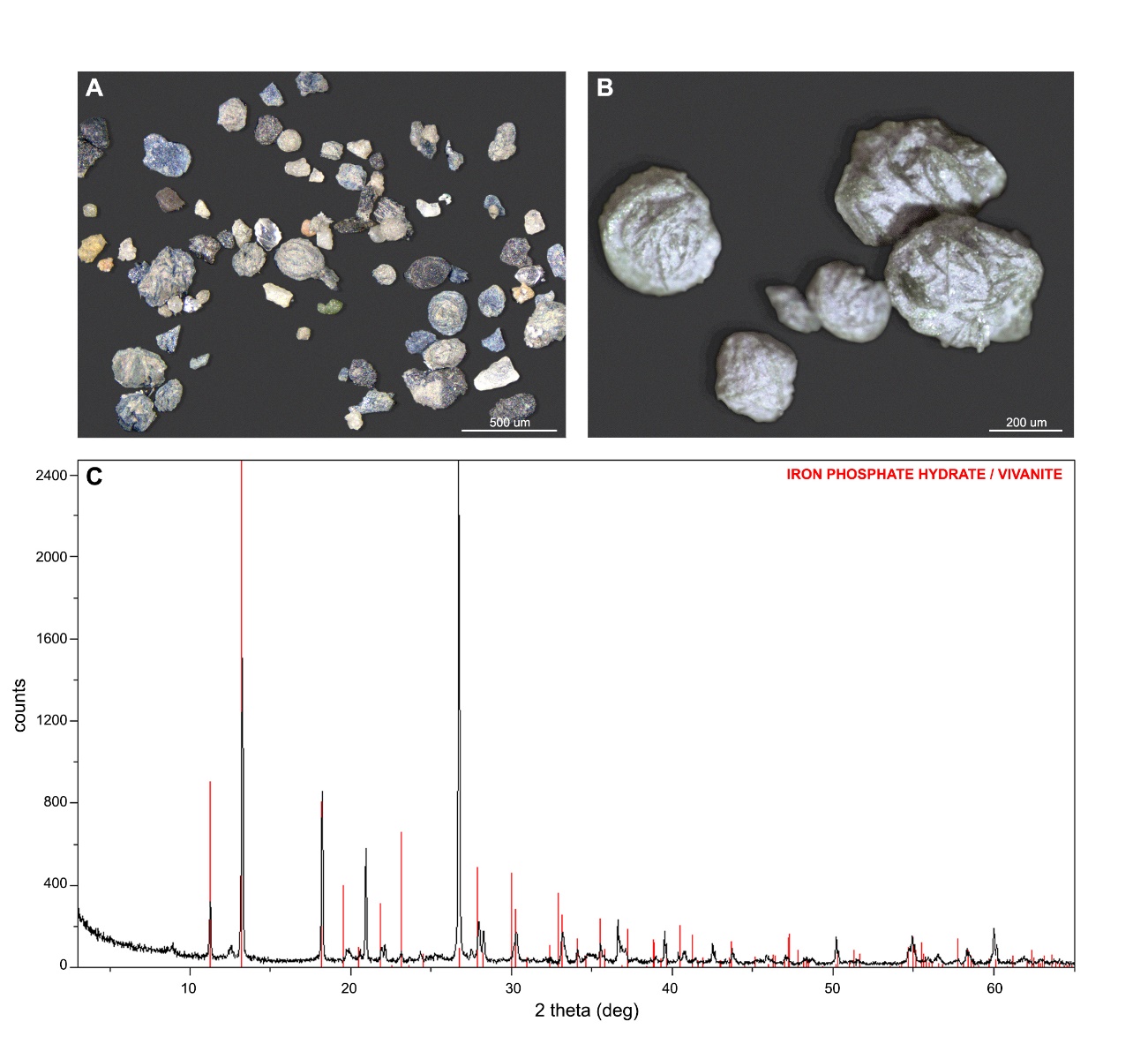
Supplementary Figure S1.** Vivianite nodules; A, B- macroscopic view of sediment and microconcretions; C- X-Ray diffraction (XRD) spectra of vivianite microconcretions.

**
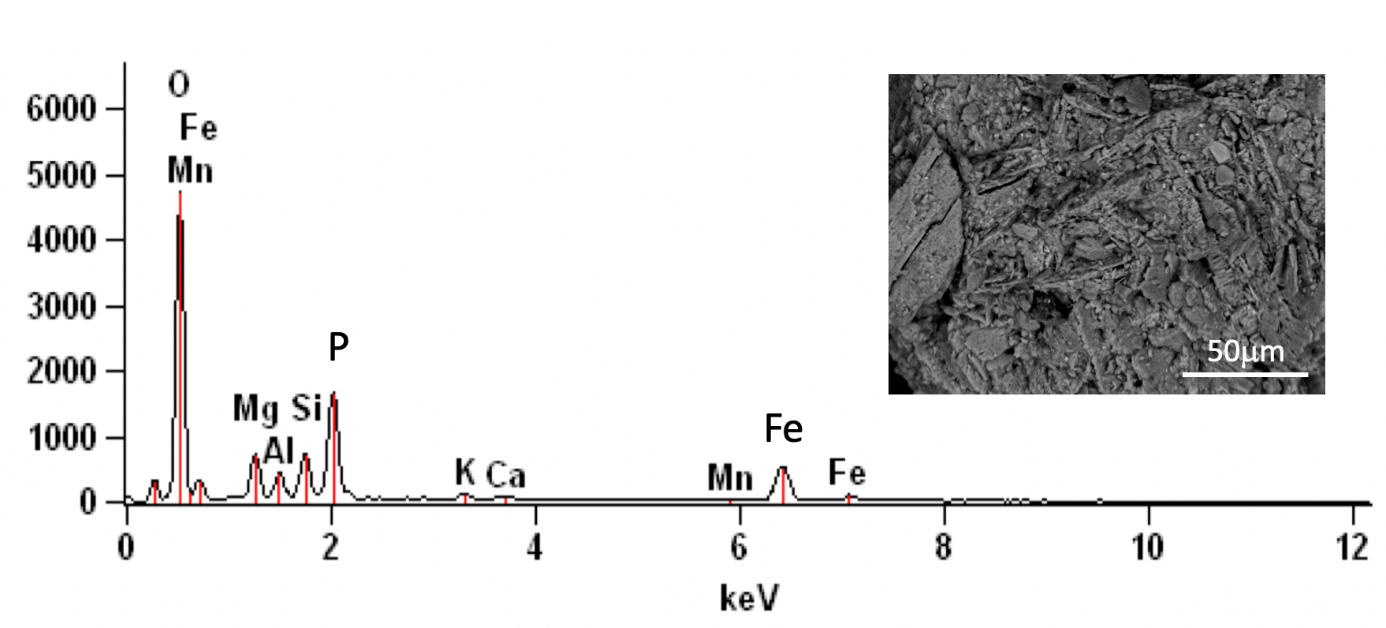
**

**Supplementary Figure S2.** EDS spectra of outer surfaces of vivianite microconcretions; vivianite with impurities of silicate indicated by small peaks of Na, Mg, Al, Si, Ca and Mn.

**
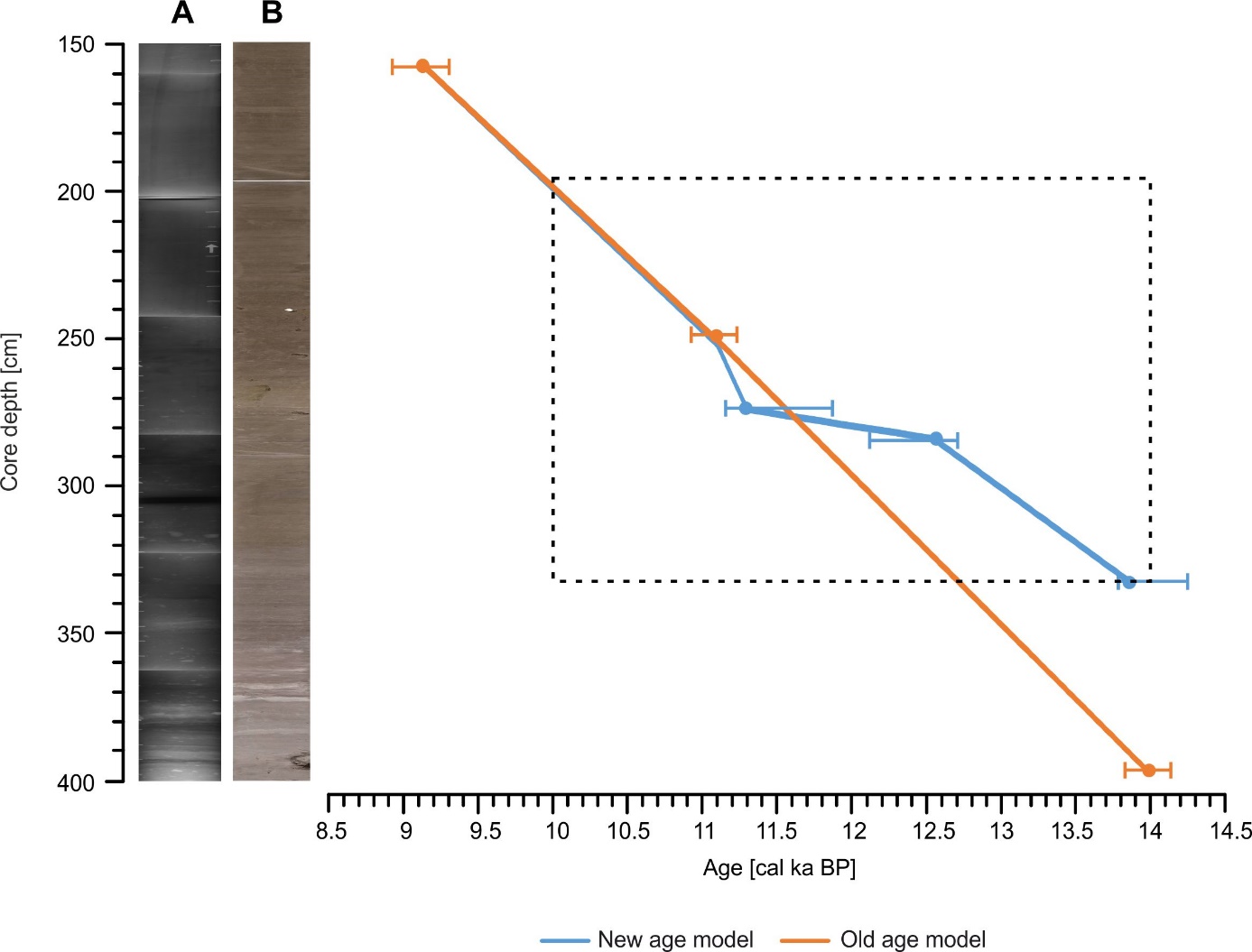
Supplementary Figure S3.** Comparison between age model of core JM09-020-GC published in Łącka et al. 2015 and the age model used in this study with 2-sigma age probability distribution indicated. On the left are (A) X-radiographs and (B) digital photographs of the core. The dashed lines mark the studied section.

**
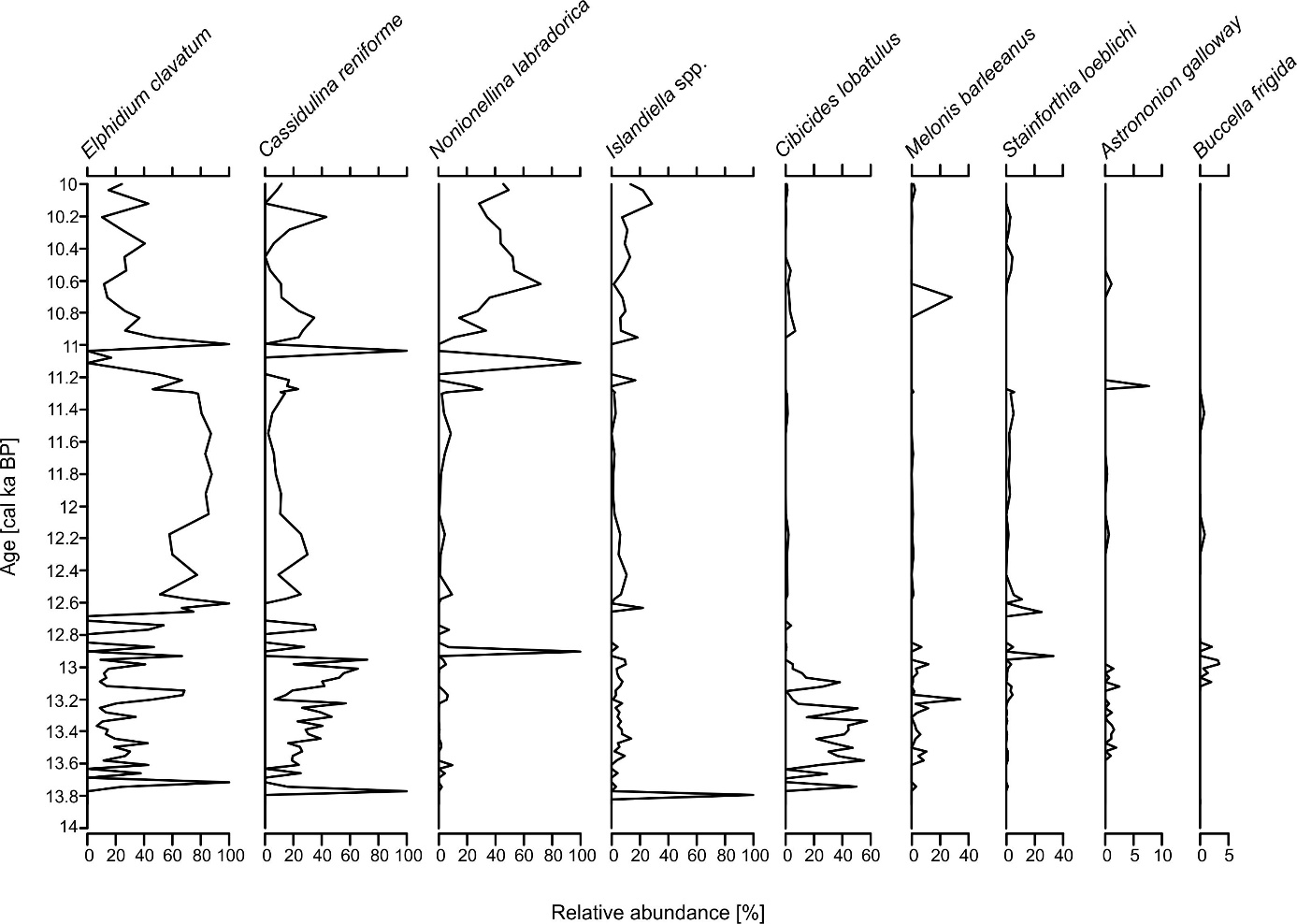
**

**Supplementary Figure S4.** Relative abundances of the 9 most common benthic foraminifera species in core JM09-020-GC.
